# Taking over Cellular Energy-Metabolism for TBSV Replication: The High ATP Requirement of an RNA Virus within the Viral Replication Organelle

**DOI:** 10.3390/v12010056

**Published:** 2020-01-03

**Authors:** Peter D. Nagy, Wenwu Lin

**Affiliations:** Department of Plant Pathology, University of Kentucky, Lexington, KY 40546, USA; roylin55555@uky.edu

**Keywords:** Tomato bushy stunt virus, yeast, plant, virus replication, virus-host interactions, glycolysis, fermentation, ATP

## Abstract

Recent discoveries on virus-driven hijacking and compartmentalization of the cellular glycolytic and fermentation pathways to support robust virus replication put the spotlight on the energy requirement of viral processes. The active recruitment of glycolytic enzymes in combination with fermentation enzymes by the viral replication proteins emphasizes the advantages of producing ATP locally within viral replication structures. This leads to a paradigm shift in our understanding of how viruses take over host metabolism to support the virus’s energy needs during the replication process. This review highlights our current understanding of how a small plant virus, *Tomato bushy stunt virus*, exploits a conserved energy-generating cellular pathway during viral replication. The emerging picture is that viruses not only rewire cellular metabolic pathways to obtain the necessary resources from the infected cells but the fast replicating viruses might have to actively hijack and compartmentalize the energy-producing enzymes to provide a readily available source of ATP for viral replication process.

## 1. Introduction

In spite of their small genomes, positive-strand (+)RNA viruses are among the most successful and wide-spread pathogens of humans, animals, and plants. Indeed, the abundant plant-infecting (+)RNA viruses only have a limited coding capacity of 4-to-12 genes. Thus, these viruses heavily depend on co-opted cellular factors and rewire several cellular pathways to support their replication in infected hosts [1,2,3,4]. Similar to animal viruses, plant viruses use viral-coded proteins together with co-opted host factors to build viral replication compartments or replication organelles (VROs) inside the infected cells. To achieve this, numerous interactions among viral components and host cellular proteins and lipids are needed that lead to their retargeting and sequestration within the VROs. Altogether, during successful infections, virus–host interactions create a subcellular environment suitable for virus replication [4,5,6,7,8].

In this review, the central player is *Tomato bushy stunt virus* (TBSV), which is the type member of the tombusviruses infecting a wide range of plants. TBSV is highly suitable for studies on virus–host interactions considering a large number of protein–protein interactions and network of interactions identified between TBSV and the host cell, based on budding yeast (*Saccharomyces cerevisiae*) [6,9,10,11].

## 2. TBSV–Host Interactions

TBSV, similar to many (+)RNA viruses of animals, induces the biogenesis of large membranous VROs, which contain numerous ~60–70 nm vesicle-like intracellular membrane invaginations with narrow openings toward the cytosol [4,12,13,14]. These small membranous invaginations harbor the viral replication complexes (VRCs) with a central role in viral RNA synthesis and the production of the new infectious progeny (+)RNAs. Whereas the TBSV p33 replication protein is an RNA chaperone that acts as the master regulator of VRC assembly [7], viral RNA synthesis is performed by the TBSV-coded p92^pol^ replication protein [15,16,17]. Similar to other (+)RNA viruses, the accessory replication protein(s), namely p33 for TBSV, orchestrates the biogenesis of the large VROs [7,13,18]. However, the biogenesis and operation of these virus-driven VROs require the subversion of a long list of cellular proteins. Moreover, VRO formation also requires major membrane deformations, membrane proliferation, and changes in lipid composition. Another critical role of VRCs is for the evasion of cellular innate defense mechanisms and protection of the viral RNA from degradation. Altogether, the discoveries made in tombusvirus–host interactions seem to be broadly applicable to many plant and animal viruses [4,7,8,14].

## 3. Budding Yeast as a Surrogate Host to Characterize TBSV–Host Interactions

One of the major challenges in current virology is the cataloging of all interactions between a virus and its host. Accordingly, the number of identified host proteins and lipids affecting various plant viruses is growing [4,7,8], but the list remains incomplete. Research with tombusviruses, which belongs to the flavivirus supergroup of (+)RNA viruses, and the unrelated *Brome mosaic virus* (BMV) and the insect-infecting Flock House virus (FHV) have taken advantage of the development of yeast as a surrogate host. This advance has allowed genome-wide screens at the single-cell level for these viruses [4,8,9,19,20]. Yeast with facile genetics has emerged as a model eukaryotic organism with conserved cellular functions and pathways. Indeed, many cellular functions and pathways have been well-described using yeast, including vesicle trafficking and secretory pathways, the actin network and microtubules, eukaryotic protein chaperones, nucleic acid and protein modifying factors, the proteasome system, to name a few. Importantly, many biochemical pathways are also conserved, including glycolysis, oxidative phosphorylation in mitochondria, protein translation, and lipid synthesis. Another advantage of using yeast as a surrogate viral host is the simple genome organization with only ~6000 genes, of which 75% have assigned functions and subcellular localization (http://www.yeastgenome.org/). Overall, the construction of various genome-wide libraries and the breadth of knowledge on yeast genes facilitates functional and mechanistic studies on virus–host interactions. In summary, yeast is an outstanding organism for system-level approaches with TBSV.

## 4. The Expanding Role of Aerobic Glycolysis

The metabolic process that converts glucose to ethanol in yeast and plants and lactic acid in animals even in the presence of oxygen is known as aerobic glycolysis or Warburg effect. In contrast, during the metabolism of healthy cells, glucose is usually converted into pyruvate, which is then channeled into mitochondrial oxidative phosphorylation in the presence of oxygen. The conversion of glucose to lactate or ethanol in the absence of oxygen is known as anaerobic glycolysis [21,22,23,24]. The aerobic glycolytic pathway is a hallmark feature of cancerous cells [21,22,23]. 

In the presence of plenty of glucose, the aerobic glycolytic pathway can quickly generate ATP at a higher rate than mitochondrial oxidative phosphorylation and provide metabolites required for anabolic processes, including the synthesis of ribonucleotides, lipids, and amino acids. The known roles of aerobic glycolysis are expanding, including healthy developmental and disease stages [21]. For example, major roles for aerobic glycolysis have been documented during mammalian retinal cell and neuronal differentiation, *Drosophila* neuroblast differentiation, and larval development [25]. *Drosophila* macrophages switch to aerobic glycolysis to fight off bacterial pathogens [26]. When activated by various stimuli, microglia in the brain increases the aerobic glycolytic pathway [27]. Additional examples of switching to aerobic glycolytic metabolism include endothelial cell differentiation, monocytes-based trained immunity, motor adaptation learning in the human brain, in rapidly dividing cells during embryogenesis, and T cell differentiation [21,22,28,29].

Aerobic glycolysis is also induced during several disease states, such as various forms of cancer, type 2 diabetes, amyloid-based brain diseases, and wound repair [28,30,31,32]. Altogether, cells and tissues utilize aerobic glycolysis as a metabolic compromise to rapidly provide ATP and new metabolic compounds for anabolic processes.

## 5. Exploitation of the Aerobic Glycolytic Pathway by Tombusviruses

TBSV replication is a rapid and robust process that requires plenty of energy in the form of ATP and molecular building blocks, which have to be produced at the sites of replication or delivered there. Accordingly, tombusviruses induce and co-opt aerobic glycolysis to produce ATP molecules within the VROs [33,34]. It has also been proposed that the co-opted aerobic glycolysis could provide ample amounts of metabolites for the cell to make molecular building blocks, such as ribonucleotides, lipids, and amino acids [22,23]. Indeed, the amounts of phospholipids, important to form new membranes, are increased by ~30% in yeast cells replicating TBSV or in infected plant cells [35]. TBSV replication also depends on new ribonucleotide and amino acid synthesis regulated by the TOR kinase cascade [36]. Whereas high glucose concentration enhances TBSV replication in yeast, 2-deoxyglucose (2-DG)-based inhibition of aerobic glycolysis reduced TBSV accumulation [36].

Why do tombusviruses need to hijack and compartmentalize the aerobic glycolytic pathway for replication? Aerobic glycolysis has many advantages over other energy-producing pathways. For example, the glycolytic enzymes are present in the cytosol, thus easily accessible for subversion by the cytosolic tombusviruses. The rate of ATP generation is higher with aerobic glycolysis than with oxidative phosphorylation within the mitochondria. Finally, aerobic glycolysis facilitates the production of molecular building blocks [22,23,37]. This allows new biomolecules to be exploited by tombusviruses to support extensive and rapid replication. One could argue that a major advantage of large VROs for tombusviruses is that it allows them to compartmentalize an entire energy-producing metabolic pathway. We also propose that aerobic glycolysis might be less exposed to feedback regulation when sequestered into the VROs than when present in the cytosol. Overall, local production of ATP within VROs might free up TBSV from the competition with cellular processes for the common ATP pool.

## 6. Exploitation of the Fermentation Pathway by Tombusviruses

**Figure 1:**
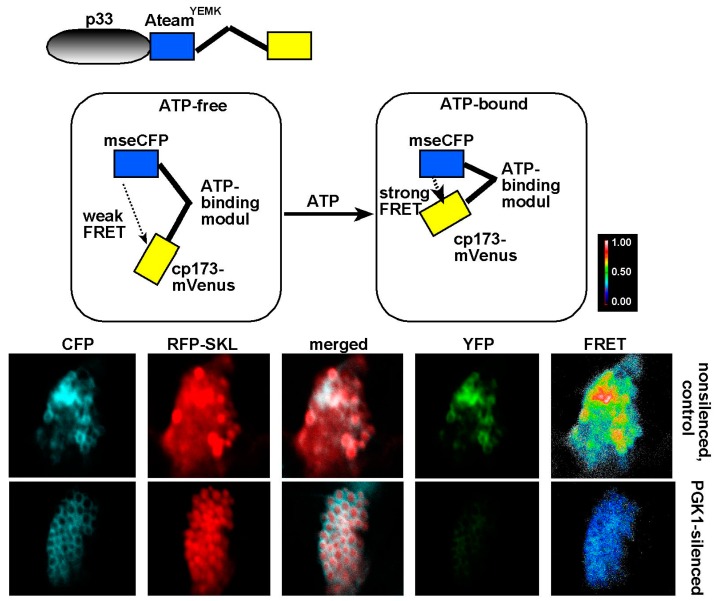
Knockdown of cellular Pgk1 glycolytic enzyme inhibits ATP accumulation locally within tombusvirus viral replication compartments or replication organelles (VROs) in *N. benthamiana*. Top: Schematic representation of the FRET-based detection of ATP within VROs. The ATP biosensor, ATeam^YEMK^ was fused to *Tomato bushy stunt virus* (TBSV) p33 replication protein. The dotted line indicates energy transfer between the modules. Bottom: Confocal microscopy images of VROs in plant cells show the low ATP level within the VRO when Pgk1 expression is silenced. Pgk1 mRNA level was knocked-down in *N. benthamiana* and the ATP level was detected via expression of the p33-ATeam^YEMK^ biosensor. The YFP signal was generated via FRET. The more intense FRET signals are white and red (between 0.5 to 1.0 ratio), whereas the low FRET signals (0.1 and below) are light blue and dark blue. *N. benthamiana* plants were infected with TBSV, which replicates on peroxisomal membranes. CFP signal detects large TBSV VRO, which is also marked by the RFP-SKL peroxisomal marker. See further details in [33].

A recent work, surprisingly, revealed efficient recruitment and compartmentalization of Pdc1 pyruvate decarboxylase and Adh1 alcohol dehydrogenase fermentation enzymes into tombusviral VROs [38]. These fermentation enzymes play critical pro-viral functions. Knockdown of Pdc1 and Adh1 in plants greatly reduced the efficiency of tombusvirus replication [38]. Enzymatically functional Pdc1 is required to support tombusvirus replication. This suggests that the role of sequestered fermentation enzymes within the VROs is to generate NAD+ from NADH. This is important for aerobic glycolysis, which requires the replenishing of the NAD+ pool. NAD+ is needed by glycolytic GAPDH glyceraldehyde-3-phosphate dehydrogenase to produce NADH, a critical regulatory step in glycolysis. By using pyruvate, the end product of the glycolytic pathway, the fermentation pathway efficiently generates NAD+ [23,37]. Altogether, our data obtained with an ATP-biosensor have shown that both glycolytic and fermentation enzymes are required for efficient generation of ATP locally within the tombusvirus VROs (Figure 1) [38]. Therefore, it seems advantageous for tombusviruses to recruit and compartmentalize both glycolytic and fermentation pathways into the VROs.

However, there seems to be an obstacle in plant leaf tissues for TBSV. This is because, unlike yeast that highly expresses the fermentation enzymes when grown in glucose-rich media, plant tissues express fermentation enzymes only at very low levels in the presence of oxygen. Therefore, tombusviruses must upregulate the expression of the fermentation enzymes in plant leaves during infection. Indeed, tombusvirus infections highly upregulate the expression of both fermentation and glycolytic enzymes in plant leaves, albeit the mechanism is not yet determined [38]. Under oxygen-poor conditions, it is known that the stabilization and relocalization of HIF1alpha transcription factor to the nucleus are required to induce the expression of aerobic glycolytic and fermentation enzymes in mammalian cells [39]. It will be interesting to learn how tombusviruses achieve this remarkable feat under oxygen-rich conditions (note that the leaves produce oxygen during photosynthesis). Another advantage for tombusviruses to hijack the fermentation enzymes is that TBSV might be able to regulate the redox potential in the vicinity of viral replication by maintaining the NAD+/NADH redox-state within the VROs.

An interesting point is that the aerobic glycolysis/fermentation pathway re-routs pyruvate, the nexus point in metabolic pathways, into the fast fermentation pathway, and away from the mitochondrial oxidative phosphorylation pathway. This then leads to the rapid regeneration of NAD+ to replenish the glycolytic pathway. NAD+ and its reduced form NADH are also necessary for the biosynthesis of nucleotides and amino acids. In general, the fermentation pathway supports fast glucose flux into metabolites and the rapid regeneration of NAD+ but, on the other hand, inhibits mitochondrial processes with unknown consequences for the host cells [22,23,37].

The dependence of (+)RNA virus replication on the glycolytic and fermentation pathways might be broad [38]. Several other TBSV-related and unrelated plant viruses have been shown to induce the fermentation pathway, thus indicating that viruses might need the rapid generation of ATP and numerous metabolic precursors for replication [38]. Since most plant, animal, and human (+)RNA viruses require the biogenesis of the membranous VROs, the local production of abundant ATP within VROs might be a widespread feature in virus-infected cells. This could open up new common antiviral strategies targeting the fermentation pathway. 

## 7. The Need for Locally Produced ATP During Tombusvirus Replication

A recently emerging picture in cell biology is that the cellular ATP pool is not readily available for intensive processes, such as cell motility or cell proliferation and rapid growth of tumor cells [40,41]. Tombusvirus replication is also a rapid and robust process occurring within the relatively inaccessible membranous VROs [13,14]. Therefore, it seems critical for tombusviruses to achieve the local production of plentiful ATP within the VROs (Figure 1). Accordingly, the robust recruitment and compartmentalization of glycolytic and fermentation enzymes into the VROs is documented in both yeast and plant cells and even in in vitro replicase reconstitution experiments [34,38]. The compartmentalization of glycolytic and fermentation enzymes within the VROs might facilitate the formation of a glycolytic metabolon. Formation of metabolons is an emerging concept on substrate channeling among enzymes of a particular metabolic pathway [42,43]. It is possible that glycolytic/fermentation metabolons, which channel substrates among the catalytic enzymes, form within the VROs to maintain rapid metabolite flux in the glycolytic pathway. This discovery may lead to a paradigm shift in virus–host interactions by putting into the limelight the need for local and efficient production of ATP within VROs in order to achieve robust RNA replication.

**Figure 2:**
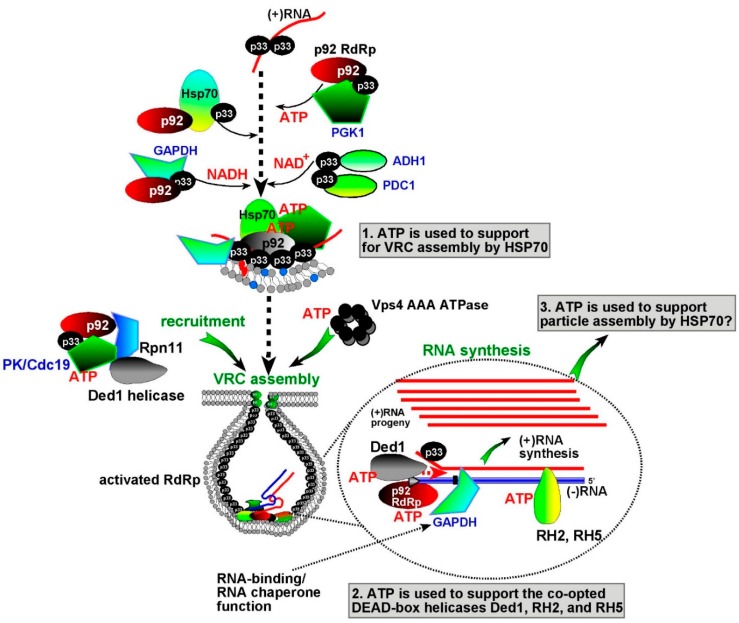
A model on the roles of the ATP produced locally in VROs in TBSV replication. Top: The early phase of replication: The newly synthesized TBSV p33 (master regulator, black circle) and p92 (RdRp, red oval) replication proteins recruit the viral (+)RNA for the pre-assembly and assembly of the membrane-bound viral replicase complex (VRC, represented by a vesicle-like structure). The co-opted glycolytic GAPDH, Pgk1, and PK and the Adh1 and Pdc1 fermentation enzymes are shown. The co-opted Hsp70 drives the pre-assembly of the VRC and the activation of the p92 RdRp in an ATP-driven manner. The ATP-dependent co-opted Ded1 and RH2/5 DEAD-box RNA helicases promote viral (+)RNA synthesis using the dsRNA replication intermediate as shown. The co-opted GAPDH has an RNA chaperone function during (+)RNA synthesis, as discussed in the text. Note, this is not the complete list of characterized host factors for TBSV [7]. The putative role of ATP in virus particle assembly is also shown.

What biochemical processes require the local high concentration of ATP within the VROs? It seems that at least two separate phases of viral replication need to be supported by ATP production (Figure 2). First, during the early steps of replication, the locally generated ATP is exploited by TBSV to support the viral replicase assembly process. We propose that the co-opted VRC assembly factors, such as the cellular Hsp70 chaperones, use ATP for their protein folding function to facilitate the insertion of the TBSV replication proteins into membranes and activate the TBSV p92 RdRp within the VROs (Figure 2) [44,45,46]. By providing high ATP concentration within the VROs, TBSV could greatly facilitate the efficiency of Hsp70-driven VRC assembly. It is also possible that additional co-opted ATP-dependent host proteins, such as the usurped actin filaments and the ESCRT Vps4 AAA+ ATPase are also fueled by the locally produced ATP within VROs [47,48,49]. This model is supported by the observation that downregulation of the ATP-generating Pgk1 phosphoglycerate kinase protein or the fermentation enzymes in host cells inhibits efficient VRC assembly and also reduces TBSV (−)RNA synthesis [33].

Interestingly, the emerging picture is that the local ATP production within the VROs is also critical during the late replication steps, including (+)RNA synthesis (Figure 2). For example, depletion of co-opted ATP-generating PK pyruvate kinase resulted in reduced (+)RNA production in yeast and plants as well as in in vitro replicase reconstitution experiments [50,51,52]. It was shown that PK provides ATP for the subverted cellular DEAD-box helicases, which are also part of the VRCs. The co-opted helicases facilitate the efficient utilization of the viral double-stranded dsRNA replication intermediates during (+)RNA synthesis in an ATP-dependent manner (Figure 2). In the case of TBSV, the (+)RNA synthesis is far more robust than (−)RNA synthesis. Whereas (−)RNA synthesis depends on co-opted RNA chaperones, such as eEF1A and eEF1Bgamma, which operate in an ATP-independent manner [53], the DEAD-box helicases depend on high ATP level during (+)RNA synthesis (Figure 2). The co-opted ATP-dependent Hsp70 chaperones are also involved in tombusvirus (+)RNA encapsidation [54], indicating that local ATP production might also facilitate virus particle assembly during replication. In summary, the current model shows that the ATP generated by the co-opted PK is used to fuel subverted RNA helicases and promote viral (+)RNA synthesis.

## 8. Do Glycolytic and Fermentation Enzymes Perform Moonlighting Functions during Virus Replication? 

The glycolytic and fermentation enzymes have been shown to participate in many noncanonical processes in cells [55,56,57]. One of the most intriguing features of glycolytic enzymes is that they all bind to RNAs [58]. Accordingly, GAPDH was found to bind to an AU-pentamer sequence on the TBSV (−)RNA, and also to the TBSV p92 RdRp protein [59,60]. These interactions are proposed to help the co-opted GAPDH act as a “matchmaker” between the viral (−)RNA and the p92 RdRp, ultimately positioning the RdRp in the vicinity of the promoter region of the (−)RNA to facilitate initiation of (+)RNA synthesis within the VRCs [60]. It is an intriguing question whether the other glycolytic enzymes might have noncanonical functions within the VROs.

## 9. Numerous Similarities Between Tombusvirus-Infected Cells and Cancerous Cells in Rewiring Cellular Metabolic Pathways 

Because both the intensive tombusvirus replication and the aggressive proliferation of cancerous cells require rapid generation of ATP and production of new biomass [23,37], there are surprisingly many similarities among virus-infected and cancerous cells. The similarities include the rewiring of the cellular metabolic pathways by shifting toward aerobic glycolytic and fermentation pathways at the expense of oxidative phosphorylation-based metabolic pathway in the mitochondria [23,37]. Accordingly, both tombusvirus-infected cells and cancerous cells upregulate the expression of aerobic glycolytic and fermentation enzymes [22,23,33,34,37,38]. These events lead to the dependence of tombusviruses and cancerous cells on high glucose concentration within the cells. Indeed, both tombusvirus replication and the proliferation of cancerous cells are highly sensitive to 2-DG inhibitor [36,37]. In addition, pyruvate, the end-product of glycolysis, has to be re-routed into the fast fermentation pathway, leading to the rapid regeneration of NAD+ to replenish the glycolytic pathway and production of either ethanol (in yeast and plants) or lactic acid (in mammals). The rapid regeneration of NAD+ allows fast incorporation of glucose metabolites into biomass [22,23,37]. Altogether, by providing abundant precursor compounds in the cytosol, the aerobic glycolytic and fermentation pathways are far more efficient to facilitate the production of molecular building blocks than the oxidative phosphorylation pathway [22,23,37]. Then, the generated new biomass can be exploited by tombusviruses to build extensive VROs and by cancerous cells for supporting rapid cell proliferation.

Another similarity emerging between tombusvirus replication and spread of cancerous cells is the need for local generation of ATP within the tombusviral VROs to support replication and for the cellular movement of cancerous cells. Indeed, it was recently documented that cancerous cells could only use the actin network for cellular movement during metastasis if aerobic glycolysis produces the ATP in the cytosol, but not by using ATP generated via oxidative phosphorylation in mitochondria [40]. 

In summary, all these cellular and biochemical similarities among tombusvirus replication, cancerous cells, and other diseases that depend on the aerobic glycolysis could facilitate cross-discipline influence of research studies and possibly the development of common cures and repurposing drugs for these diseases.

## 10. The Role of Glycolysis in Virus–Host Interactions

Many viruses are known to activate and reprogram cellular metabolism, including glycolysis [61]. Metabolic profiling of primary human cells infected with Dengue virus revealed that virus replication induces the upregulation of glycolytic enzymes and the glucose transporter 1. The increased glucose consumption by the host cells was required for optimal Dengue virus replication [62]. Global metabolic profiling was also used to show enhanced glycolysis in cells infected with Kaposi’s Sarcoma-associated herpesvirus (KSHV) [63]. Proteome profiling of hepatitis C virus (HCV)-infected cells unraveled the upregulation of several glycolytic enzymes, suggesting significant perturbations in cell metabolism [64]. Accordingly, the activity of the glycolytic hexokinase is increased after interaction with NS5A replication protein of HCV [65]. HCV induces aerobic glycolysis via activating hypoxia-inducible factor 1 (HIF-1), which is the master regulator of this metabolic pathway [66]. Other viruses, such as vaccinia virus, Epstein–Barr virus and KSHV, stabilize HIF-1 to promote aerobic glycolysis [67,68,69]. The activity of the ATP-generating pyruvate kinase (M2 isoform, PKM2) is enhanced by phosphorylation by Src protein of Rous sarcoma virus [70]. Oncogenic viruses induce aerobic glycolysis and lactate production during their latent infections [30,63]. For example, hepatitis B virus activates the mTOR signaling cascade, resulting in induction of the aerobic glycolysis pathway [71]. Additional examples with several oncogenic virus-driven regulations of the aerobic glycolytic pathway can be found in a recent review [30].

## 11. Future Directions

The active hijacking of the glycolytic and fermentation enzymes by TBSV into VROs opens up many significant areas for further research in the future. What is the actual mechanism of the hijacking of the glycolytic and fermentation enzymes by TBSV? What are the roles of catalytic versus RNA-binding functions of the glycolytic and fermentation enzymes during viral infections? How broad is this phenomenon among eukaryotic viruses of plants, fungi, insects, animals, and humans? It is possible that fast replicating viruses that reach high titers and produce abundant progeny viruses might need the local production of ATP within the VROs. How could the infected cells cope or even respond to the lesser availability of the hijacked enzymes and/or reduction of the cytosolic ATP pool? Thus, what are the consequences for the various types of cells when they face this challenge? Is it possible to exploit the common strategies of viral replication to develop broad-spectrum antivirals? Future studies could answer if it might be possible to repurpose anticancer or anti-inflammatory drugs targeting various glycolytic enzymes or their regulators as antivirals [72].

## 12. Conclusions

Incapable of producing their own energy supply, (+)RNA viruses must usurp ATP from the host cells to fuel the energy requirement of viral replication. As a new paradigm shift, tombusviruses achieve the production of ATP locally within VROs due to virus-driven hijacking and compartmentalization of both the cellular glycolytic and fermentation pathways. This allows TBSV to provide ATP for RNA virus replication locally without directly competing with the host cell for the cytoplasmic ATP pool. Why is compartmentalization of the aerobic glycolytic and fermentation pathways in the VROs advantageous for tombusviruses? The combined subversion of the aerobic glycolytic and fermentation pathways allows for the rapid production of ATP locally, including replenishing of the regulatory NAD+ pool by the fermentation pathway. Then, the locally produced ATP could be used efficiently by the co-opted ATP-dependent host factors required for pro-viral processes [33,34]. This benefits VRC assembly, the activation of p92 RdRp, and the utilization of both ssRNA templates and dsRNA replication intermediates for viral RNA synthesis [7,33,34]. The local production of ATP within the VROs might also be necessary for other viruses to avoid direct competition with cellular processes for the common ATP pool. Moreover, all the molecular processes could be accelerated by the high local concentration of ATP within the VROs. Rapid replication by viruses might allow them to speed ahead of antiviral responses of the hosts and outcompete other pathogenic viruses. It is also possible that the feedback regulation of these metabolic processes by the cell is less efficient when compartmentalized in the VROs. The gained knowledge of co-opted host factors could lead to novel, inducible, broad-range, and durable antiviral tools against plant and possibly animal viruses.
